# Training supported by simulated persons to promote the development of specific communication skills in advance care planning

**DOI:** 10.3205/zma001735

**Published:** 2025-02-17

**Authors:** Kornelia Götze, Stefanie Otten-Marré, Barbara Loupatatzis, Jürgen in der Schmitten

**Affiliations:** 1 University of Duisburg-Essen, Medical Faculty, Institute of Family Medicine & General Practice, Essen, Germany; 2 University Hospital Düsseldorf, Centre for Health and Society (chs), Institute of General Practice & General Practice, Düsseldorf, Germany; 3 GZO Wetzikon Hospital, Palliative Care Team, Wetzikon, Switzerland

**Keywords:** teaching methods, simulation patient/person, training for complex communication skills, advance care planning

## Abstract

**Objectives:**

Advance care planning (ACP) has been conceived to ensure that patients who are unable to consent are treated in consistence with their well-informed, predetermined preferences. At an individual level, standardised conversations are offered by specifically qualified healthcare professionals (ACP facilitators). Internationally, there is considerable variability with regard to ACP qualifications. This article describes how ACP facilitators are trained in role plays employing simulated persons (SPs) in accordance with the standards of two professional societies.

**Methods:**

ACP experts developed ten roles in cooperation with an SP coach (director) based on real ACP conversations. The emotional and mental world of the role, ACP-relevant facts such as previous experience in the medical context, and aids for acting were developed, taking into account the central challenges in ACP conversations. To ensure standardisation, the SPs rehearsed in a structured manner and received feedback training. Microteaching techniques were developed for the facilitator training.

**Results:**

Feedback skills and openness towards the topics of serious illness, dying and death are required for the SP to be suitable. Since 2017, ACP facilitator training has taken place in small groups of four participants, one SP and one qualified ACP coach. The required framework is described in detail.

**Conclusion:**

SP-supported training is a decisive improvement for the teaching and assessment of the highly complex ACP-facilitation skills and attitudes. Planning and implementation place high demands on SP and ACP coaches who also require specific qualifications.

## Zusammenfassung

**Zielsetzung:**

Advance Care Planning (ACP) kann gewährleisten, dass einwilligungsunfähige Patient*innen gemäß ihren wohlinformierten vorausverfügten Präferenzen behandelt werden. Auf individueller Ebene werden hierfür standardisierte Gespräche durch qualifizierte Fachpersonen (ACP-Gesprächsbegleiter*innen (GB)) angeboten. Bezüglich der GB-Qualifizierung besteht international erhebliche Variabilität. In diesem Artikel wird dargestellt, wie ACP-GB gemäß den Standards zweier Fachgesellschaften unter anderem mit Hilfe von Simulationspersonen (SP)-gestützter Trainings qualifiziert werden.

**Methoden:**

ACP-Expert*innen haben in Anlehnung an reale ACP-Gesprächsbegleitungen mit einer SP-Trainerin (Regisseurin) zehn Rollen entwickelt. Unter Berücksichtigung zentraler Herausforderungen in ACP-Gesprächen wurden die Gefühls- und Gedankenwelt sowie ACP-relevante Fakten wie Vorerfahrungen im medizinischen Kontext der Rolle und schauspieltechnische Hilfestellungen erarbeitet. Für eine Standardisierung wurden die SP strukturiert eingeprobt und erhielten eine Feedbackschulung. Für den Unterricht wurden Micro-Teaching-Techniken entwickelt.

**Ergebnisse:**

Für die Eignung der SP sind Feedback-Kompetenzen sowie Offenheit gegenüber den Themen schwere Erkrankung, Sterben und Tod erforderlich. Seit 2017 wird in Kleingruppen mit je vier Teilnehmenden, einer SP und einer hierfür qualifizierten ACP-Trainer*in unterrichtet. Die hierfür erforderlichen Rahmenbedingungen werden im Artikel detailliert beschrieben.

**Schlussfolgerung:**

SP-gestützte Trainings sind für die Erlernung und Bewertung der hochkomplexen ACP-GB Kompetenzen und ihrer Haltung ein entscheidender Zugewinn. Planung und Durchführung stellen hohe Anforderungen an die SP und ACP-Trainer*innen, die ebenso spezifischer Qualifikation bedürfen.

## 1. Introduction

### 1.1. Advance care planning

Advance care planning (ACP) aims to ensure that patients who are unable to consent [1] receive care consistent with their well-informed predetermined preferences [2], [3]. This requires comprehensive developments in the healthcare system at an individual, institutional and regional level (ACP programme) [4]. The institutional and regional implementation of the ACP programme must ensure that the preferences of the person incapable of decision-making are known and honoured in the event of severe illness. At the individual level, a multi-stage outreach offer to have an ACP conversation is made by a qualified specialist, a so-called ACP facilitator. The conversation can lead to a meaningful advance directive.

### 1.2. ACP facilitator qualification meeting the standards of ACP Germany and ACP Swiss

**Figure 1:**
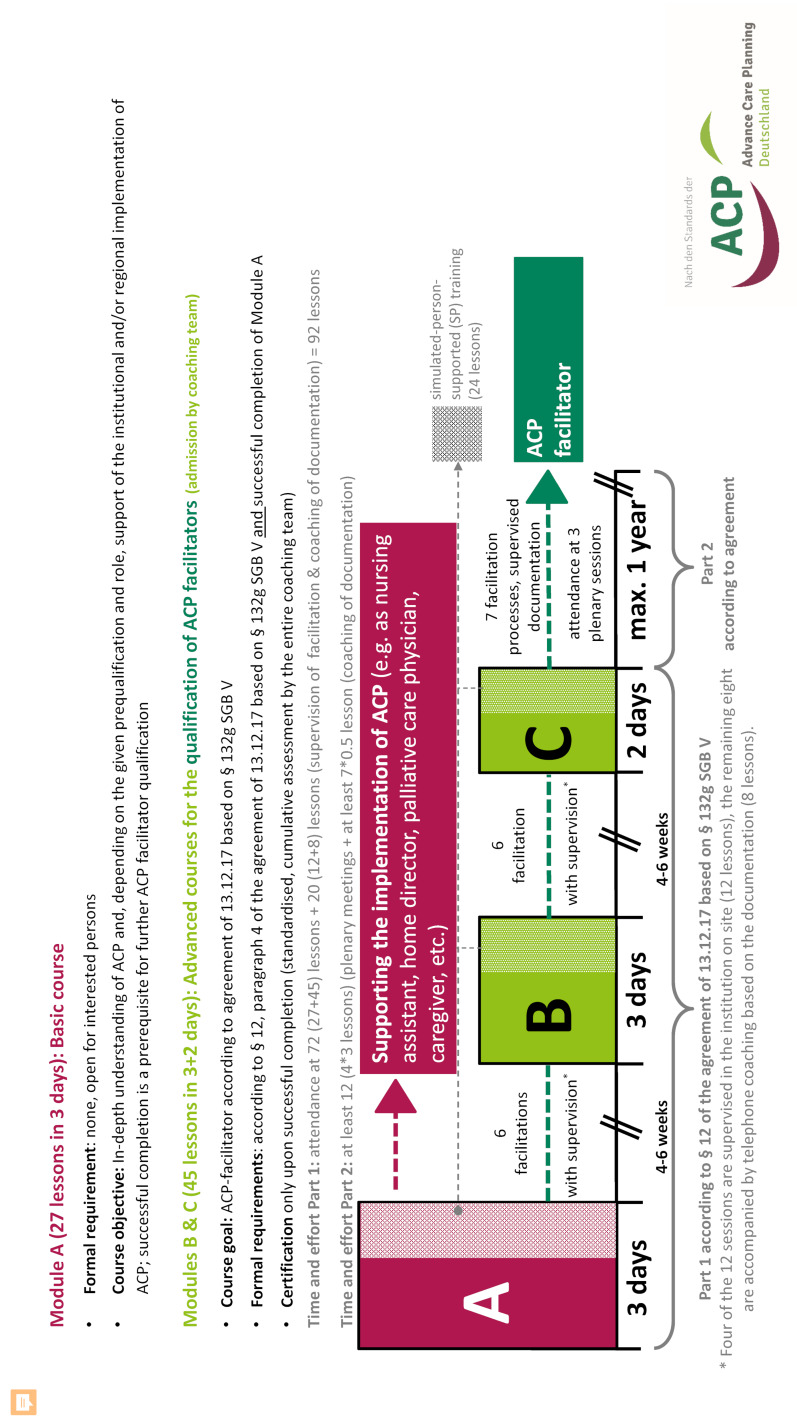
Overview of the 8-day ACP facilitator qualification from ACP Deutschland e.V. following the introduction of SP-supported training in 2017. Further details of the ACP facilitator qualification, such as the daily schedule, can be found in Götze et al. 2022 [8], additional file 1. Before the introduction of SP training, there were already participants and demonstration role plays (see table 1). With the introduction of the SP-supported training, the course was divided into three modules in order to intensify the facilitation between the modules in practical application. ACP: advance care planning, TU: teaching units (45 minutes), SGB: German Social Security Code, SP: simulated person. This figure was originally published in Götze K et al. 2022 [8], additional file 1. © Götze et al. https://creativecommons.org/licenses/by/4.0/

Internationally, there have been training programmes for ACP facilitators since the 1990s [5], but only a few of these have been implemented with simulated persons (SP) (e.g. to tatou reo – New Zealand [6]); there is no systematic survey on this subject. The joint standard of the professional societies ACP Germany (ACP-D) [7] and ACP Swiss [8] for an eight-day ACP facilitator qualification, on which this article is based, was developed in 2017/18 on the basis of the respective national predecessors [7], [9], [10], [11] and internationally established ACP programmes [5], [12] (see figure 1 (Fig. 1) and attachment 1 ). The standard draws from experience in SP-supported teaching “breaking bad news” to medical students with similar requirements regarding communication skills [13], [14].

The four sections in the ACP conversation described below are first introduced theoretically during the ACP facilitator qualification and then practiced with SP and in real life (see attachment 1 , table S1b).

ACP facilitation for the planning of treatment goals and possible limits in future life-threatening situations in advance touch on sensitive and existential issues [15], [16]. Therefore, exploration of the individual's underlying treatment goal by discussing personal “attitudes towards life, dying and serious illness” form the basis for the medical preferences in the ACP process. Beyond that, specific care preferences are determined for acute emergencies [17], for hospital treatments in case of decisional incapacity of uncertain duration [18], and for treatments in case of permanent loss of decision-making capacity. For persons incapable of decision making the surrogates are empowered to record the presumed care preferences of the person concerned for emergencies and for the event of a future deterioration of their condition [8], [19] (see attachment 1 , table S1b).

Conducting the conversation requires a combination of a particularly sensitive, open-ended approach and a certain degree of lead so that the people engaged in advance care planning can clarify the key issues for themselves. This sensitive approach can include, touching on taboo subjects as well as enduring and carefully channeling the associated strong emotions in order to enable valid advance care planning [20].

The teams dealing with a medical crisis must also be able to rely on the fact that the documentation of these conversations is a reliable reflection of the person's care preferences (validity). By implication, this means that the participants should only pass the qualification and be certified accordingly if the required skills (see attachment 2 ) have been confirmed by a reliable skill and performance assessment (see attachment 3 ) as part of the ACP facilitator qualification.

### 1.3. SP-supported training to improve communication skills

Role-play is ideal for deepening communicative skills since these cannot be reliably built up through mere listening or interactive learning [21]. The strength of participant role-play lies in the change of perspective. The additional use of SP-supported role-play in the ACP facilitator qualification (see table 1 (Tab. 1) and table 2 (Tab. 2)) makes possible

- to reliably anchor standard situations in the ACP facilitation process (especially challenges) in the curriculum [22],
- to include the option of going back to a specific point in the conversation with pinpoint accuracy in order to repetitively practice particularly challenging aspects of conducting the ACP conversation [23],
- to give participants a sense of achievement [23], and
- to perform a cumulative skills assessment with a standardised level of difficulty (see table 2 (Tab. 2)) [24].

When training communicative skills, SPs portray people in various scenarios [13]: from short medical histories to complex, emotionally charged conversational situations in which the attitude, preferences, biography and fate of the presented patient-role may be revealed [25].

As different as the simulated conversations are, the acting challenges for the SP also vary. While the simpler conversations require the most accurate possible recapitulation of the role's medical history as well as flexible interaction with and spontaneous reactions to the other person, more complex conversational situations require, for example, the truthful portrayal of emotions and the inner attitude of the role [26]. In addition, corresponding body language must be used and the conversational situations to be called up must be repeatable and standardised [26].

The scripts of the respective role and the rehearsals of the SP must be designed accordingly. In roles that are intended to train more complex communication skills, medical details take a back seat to life story, thoughts and emotions and only form a thematic framework for the respective role [27]. Moreover, since the trained SPs are medical laypersons, it would be unhelpful to focus on medical details in the role script. Rather, inner images and memories of patients regarding their symptoms and illnesses as well as challenges and effects in everyday life give the actors a deeper impression of the existential framework and the experience of the illness from the perspective of the roles [26].

Another strength of SP-supported training is the professional feedback provided by well-trained SPs from the perspective of the role directly to those conducting the conversation [13]. It is very impressive to receive a description of how the patient perceived the conversation from the experience of the patient role and what the SP would have wished for in the role. This can support the participants’ self-reflection.

## 2. SP-supported training in the qualification of ACP facilitators

The following is a detailed description of SP-supported training, from the conception of the roles and rehearsal work to the organisation of the course and the actual implementation of the SP-supported training.

### 2.1. Eligibility of the SP

Professional actors and trained laypersons, some of whom already have experience as SPs in teaching, are employed for SP-supported training as part of the ACP facilitator qualification. In order to develop eligibility criteria for SPs, comparative observations and structured exchanges were conducted in the context of five ACP facilitator qualifications in 2017 and 2018 by ten ACP coaches and three SP coaches [28]. There were no indications that the professional actors were better able to cope with the requirements than were the well trained laypersons.

Sometimes the professional actors were able to access emotions and body language more easily and develop the role more quickly. For a good performance as an SP, however, spontaneous interaction with the participant and the ability to give detailed feedback are crucial in addition to classical acting. An openness to allow, endure and reflect on the taboo subjects of death and dying has proven to be central to these interactions. This openness is related more to personality than to expertise as an actor.

### 2.2. Conception and rehearsal of the roles

Phenomena and challenges frequently encountered in real ACP faciliations were identified for the design of the roles in order to train them under secure course conditions with the aim of being able to put what has been learnt into practice in new and modified everyday situations [22]. Ten roles were derived based on real people engaged in advance care planning conversations (see attachment 4 ).

Comparatively extensive (~ 10,000 words) role scripts were developed for the ten characters by ACP coaches in collaboration with an SP coach.

The structure of the script was based on the course of the standardised ACP conversation. In the role scripts, the biography and the emotional, life and mental situation of the persons to be simulated were formulated in general as well as attitudes, opinions, previous experiences, thoughts and feelings that are of particular importance for the topics of advance care planning.

**Figure 2:**
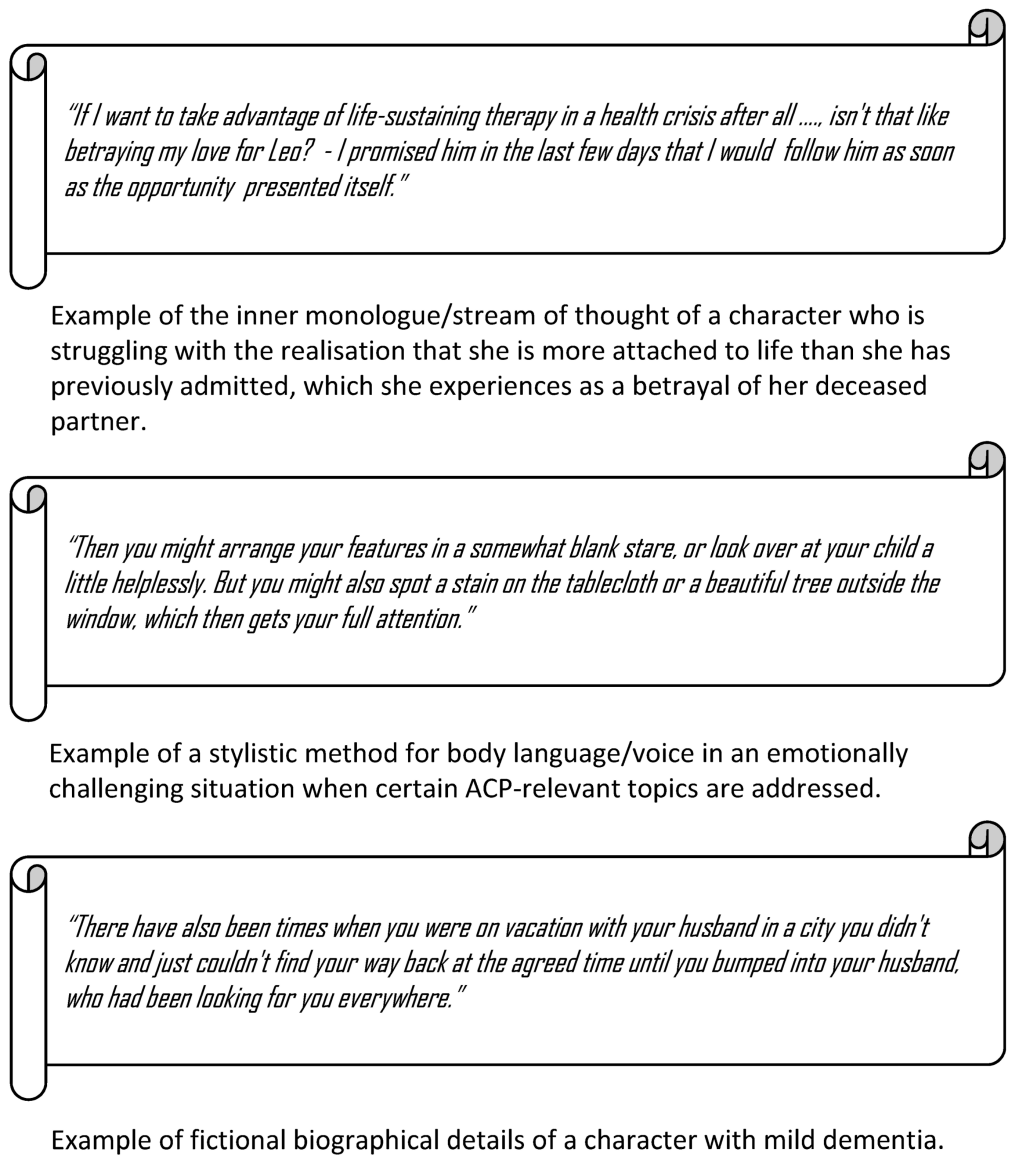
Exemplary sections from the role scripts for the simulated persons in the ACP facilitator qualification

In order to facilitate access to the role, various technical acting aids were included in the role scripts. These include, for example, inner monologues and non-verbal and verbal cues such as posture or voice modulation (see figure 2 (Fig. 2)).

The scripts were refined in an iterative process as part of the rehearsal work and SP performances in order to ensure standardisation when incorporating future SPs (see table 2 (Tab. 2)).

Facts relevant for advance care planning (e.g. illnesses experienced by oneself or others) or medical facts were combined with their subjective significance for the particular role in order to achieve the most homogeneous interpretation possible by different SPs. This detailed role representation permitted a high degree of standardisation of the roles, even over long periods of time (see table 2 (Tab. 2)).

#### 2.2.1. Preparation for the rehearsal

Ten SPs from the SP pool established for teaching purposes at Heinrich Heine University in Düsseldorf (CoMeD) were rehearsed for the ACP facilitator qualifications. The SPs were briefed in advance about the project and the task to be completed; in particular, the sensitive topics of illness, death and dying were discussed with the SPs in this protected setting. The role script, which was sent out well in advance of the rehearsals, was prepared independently by the SPs. All SPs, the SP coach and at least one ACP coach were requested for the rehearsal days. Half a rehearsal day per role proved to be useful.

#### 2.2.2. On-site rehearsal of the SP

During the rehearsals, there was an exchange and reflection on the respective role. The group created an identity card (IC) for the roles of the persons undertaking an advance care planning conversation on a flipchart, on which the most important character traits were recorded as well as possibilities for translation into body language and expression [29]. This IC is a concise summary of the role and its portrayal and has since supported the SPs in evoking the character during the ACP facilitator courses. The core component of the rehearsals was practicing the role in the conversation. For this purpose, an ACP coach was available to support the SPs as a conversational partner. The performance was then reflected on together. Additions and corrections were discussed and recorded in the script and the IC. In addition, acting assistance was given for the performance or for getting into and out of the role, and giving feedback after the conversation with the SPs was practised.

Joint development and rehearsal of the roles improved the standardisation (see table 2 (Tab. 2)) since the SP colleagues served as orientation [30].

### 2.3. Organisation before and during the ACP workshops with SP

When planning the ACP facilitator qualification, additional time-consuming tasks must be taken into account when adding SPs, for which special resources must be planned. For example, SPs must be contacted and coordinated so that one SP is available for each small group of four participants at the appropriate times in addition to the ACP coach. In addition, an SP coach should be available to the SPs during and after the assignments for questions about the roles and for debriefing, even at short notice, at least by telephone or online.

Rotation plans drawn up in advance by the ACP course centres can ensure that the participants ideally always experience the same SP in the same roles in the course of the qualification, as the roles are accompanied by several ACP conversation sections during the course. The level of difficulty also increases from role to role depending on the ACP conversation section to be trained. In these rotation plans, the SPs are clearly assigned to the rooms and people in order to ensure that the course runs smoothly.

Prior to the course, the SPs receive a letter containing all relevant information on times, locations and rotations.

If possible, the SPs should be provided with a separate room on site for preparation, props and breaks. They can prepare there and get into and out of their roles after their performances. This retreat room also helps to separate the real people (SPs) from the roles that the SPs embody for the participants.

With regard to the SPs, it should be borne in mind that the simulations are a challenge for many participants, especially at the beginning, e.g. situations they have experienced themselves that were characterised by serious illnesses or the loss of loved ones can become present again through the simulation of the ACP conversation, as we have often experienced in the courses. Experience has shown that these challenges for the participants in the new role of ACP facilitator can sometimes lead to emotionally charged (possibly even hostile) behaviour towards the SPs, which can be stressful for everyone. In our experience, special attention by ACP and SP coaches is therefore necessary to enable these situations to be worked through not only with the participants, but also with the SPs. It has proven to be wise to offer the SPs a debriefing (see above) with an ACP coach with SP experience from the course and the SP coach (by telephone/online if necessary) directly after their performance. Experience has shown that continuous quality assurance, provided by SP coaches supporting the ACP facilitator courses on site, is necessary after two to three performances, at new course centres, and during the introduction of new SPs or new roles [13] in order to maintain standardisation.

### 2.4. Implementation of the SP-supported teaching lessons

The SP role-play takes place in small groups of a maximum of four participants, with each group guided by a qualified ACP coach.

Before the SP-supported training begins, the sitting positions, i.e. the setting, are organised. It is essential that the participants are actively encouraged by the coach to adjust their own sitting positions and those of the simulated person(s) engaged in an advance care planning conversation at their own discretion and to place the ACP coach according to their preferences. Through this leeway and the associated responsibility, the participants learn to develop an awareness of the importance of the setting. Both the needs of the ACP facilitator and those of the planning person (client) need and deserve to be considered. The other participants are given observation tasks (e.g. observing verbal and non-verbal communication).

After clarifying any open questions from the participant who is now conducting the ACP conversation, the available time and the rules of microteaching, the simulation begins. With a clear, pre-agreed signal, the coach has the option of interrupting the conversation for a microteaching unit. During the interruption, the SPs have the task of preserving their emotions from the previous minutes of the conversation as far as possible so that the simulation can be resumed seamlessly at a point determined by the coach and participants.

Before continuing the simulation, the ACP coach ensures that the SP is aware of the (new) starting point and is ready. This is often done non-verbally. The simulation is continued at a clear signal from the coach.

At the end of a simulation, the participants are debriefed and the SP and coach give feedback to the participants. The observations of the other participants are included in the feedback.

#### 2.4.1. Microteaching

Microteaching (also described as Rapid Cycle Deliberate Practice [31]) in the sense used here means working closely on the course of the conversation, what is said and what is perceived; there are more frequent interruptions instead of focussing on the flow of the conversation and feedbacking a wrap-up at the end. This helps to prevent a cumulating list of detailed feedback especially in the case of inexperienced ACP facilitators, the processing of which would be overwhelming and remain incomplete. Above all, the classic two-part role-play format (=longer simulation phase followed by critical discussion) lacks the opportunity to directly try out alternative behaviour for a specific situative detail and possibly experience a sense of achievement.

The technique of microteaching has proven its worth in order to make optimum use of the limited time available for simulation training and to give participants both a feeling of self-efficacy through successful repetition and the steepest possible learning curve. Microteaching also makes it possible to identify participants who find the task of ACP faciliation too challenging or for whom it is not suitable as part of the skills assessment and self-reflection.

On the other hand, microteaching has the disadvantage that the flow of conversation is repeatedly interrupted; in our experience, participants can occasionally become so irritated by this that they are no longer able to continue the role-play. It is the task of the ACP coaches to recognise such irritation as early as possible and to make use of the didactic instrument of microteaching (see table 3 (Tab. 3)) judiciously. In individual cases, an unscheduled switch of the participant in the role of ACP facilitator can be useful in order to relieve the participant irritated by the interruptions.

The techniques listed in table 3 (Tab. 3) have become established in the ACP facilitator qualifications for organising microteaching, and go beyond the techniques known to the authors from the literature [31], [32].

### 2.5. Standardisation during the creation of multiple SP pools

After the first SP pool for ACP facilitator qualifications meeting ACP-D standards was established in Düsseldorf, it became necessary to create SP pools for ACP facilitator qualifications at other ACP facilitator course centres (including centres in Zurich, Munich, Frankfurt, Göttingen and Marburg) as demand for courses increased. The testing of the local SPs for the roles was carried out in mutual consultation with the support of the Düsseldorf SP coach (co-author of this article: SOM) with the aim of transferring the know-how developed in Düsseldorf and realising a Germany-wide standardisation of the SPs (see table 2 (Tab. 2)) and thus also of the ACP facilitator qualification [30]. The regional SP coaches are responsible for coordinating and monitoring the performances as part of the ACP facilitator qualification.

## 3. Conclusions

SP-supported trainings are a decisive improvement for the teaching and evaluation of the highly complex ACP facilitator skills and their attitude. In addition, according to the many years of experience gained by coaches certified by ACP Germany or ACP Swiss, it cannot be replaced by any other methodological format for this particular qualification. Developing the roles, selecting and testing the SPs, organising the assignments and conducting the simulation training in small groups places high demands not only on the SPs, but also on the ACP coaches in particular, who require their own specific qualifications in this regard [8], [33], [34].

## Notes

### Authors’ ORCIDs

- Kornelia Götze: [0000-0001-8134-7521]
- Stefanie Otten-Marré: [0009-0002-7064-6109]
- Barbara Loupatatzis: [0009-0006-7276-5944]
- Jürgen in der Schmitten: [0000-0001-5960-1511]

### Funding

The development of the SP training was funded by the Federal Ministry of Health as part of the project “Promotion of the implementation of §132g SGB V according to HPG” under the reference no. ZMVI1-2516FSB801.

### Secondary publication

This manuscript is also published in a slightly adapted version and without figures, tables and supplements in Praxisbuch Advance Care Planning. Behandlungsentscheidungen gemeinsam vorausplanen (in print) [35].

## Acknowledgements

I. Karzig, D. Otto and Prof. Dr. T. Krones from the ACP working group of University Hospital Zurich as well as Prof. Dr. Dr. B. Feddersen, Prof. Dr. G. Marckmann and Dr. S. Petri from the ACP Germany working group “qualification”, with whose support the qualification for ACP facilitation was designed. Prof. Dr. H. Stanze for her support in writing a role script (inclusion assistance). All other ACP coaches who carry out the ACP facilitator qualifications according to these standards.

## Competing interests

Barbara Loupatatzis, Jürgen in der Schmitten and Kornelia Götze are ACP coaches and received fees for conducting ACP qualifications, lectures or workshops on ACP. Jürgen in der Schmitten was (2011-2021) and Kornelia Götze is (since 08/2021) treasurer of the international professional association Advance Care Planning international e.V. Jürgen in der Schmitten (since 02/2017) and Kornelia Götze (since 06/2021) are board members of Advance Care Planning Deutschland e.V. Barbara Loupatatzis receives fees for the qualification of SPs in the context of ACP-Swiss e.V. qualifications. Stefanie Otten-Marré receives fees for the coordination and qualification of SPs as part of ACP qualifications. Translated with DeepL.com (free version)

## Supplementary Material

Course descriptions

Competencies of ACP facilitators according to structural, process and outcome criteria

Competence and performance evaluation form according to the standards of ACP Germany

Description of the challenges of the simulated persons (SP) roles and most relevant training objectives for the ACP facilitator qualification

## Figures and Tables

**Table 1:**
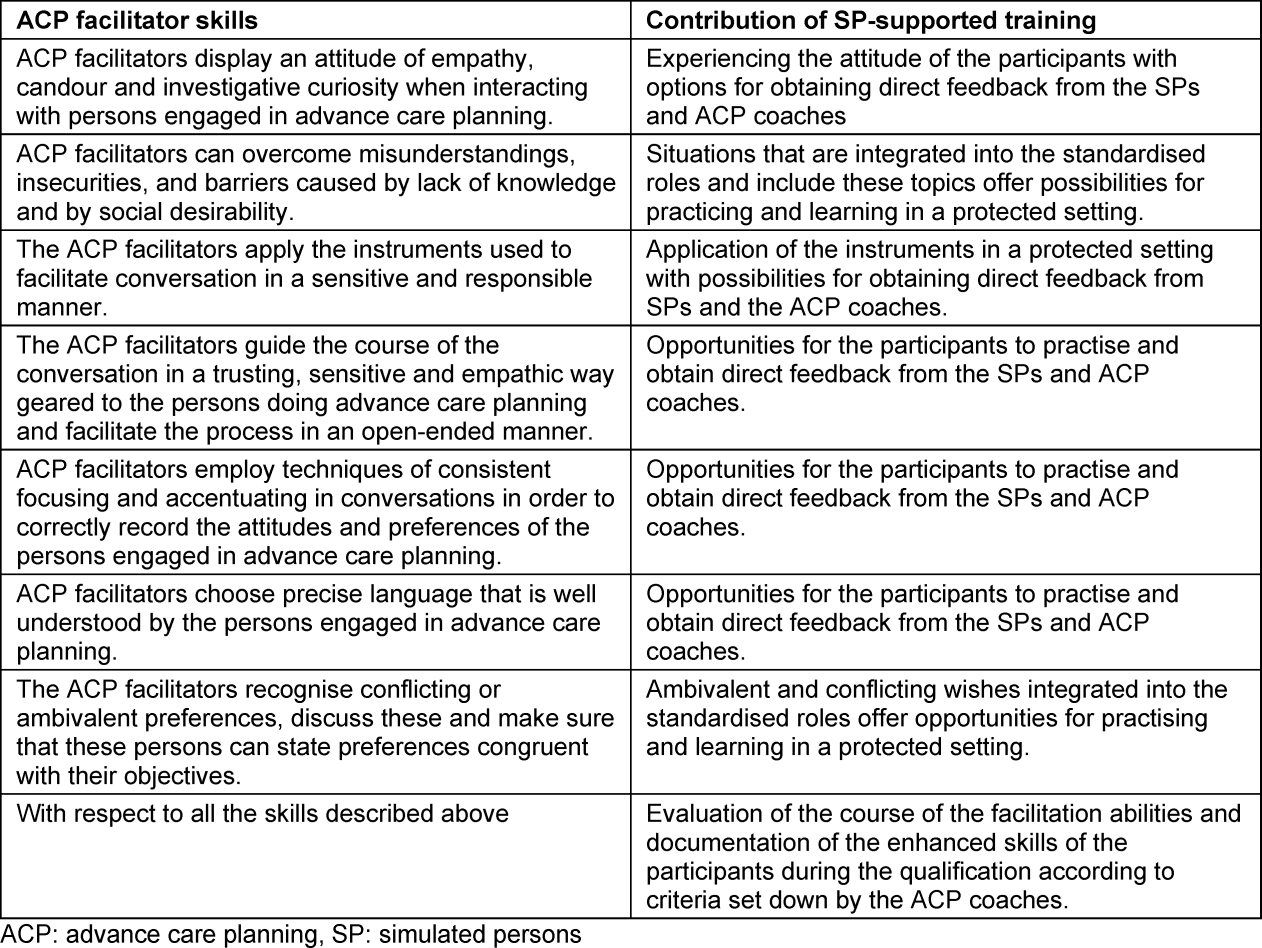
Role of SP-supported training in the acquisition of ACP facilitator skills

**Table 2:**
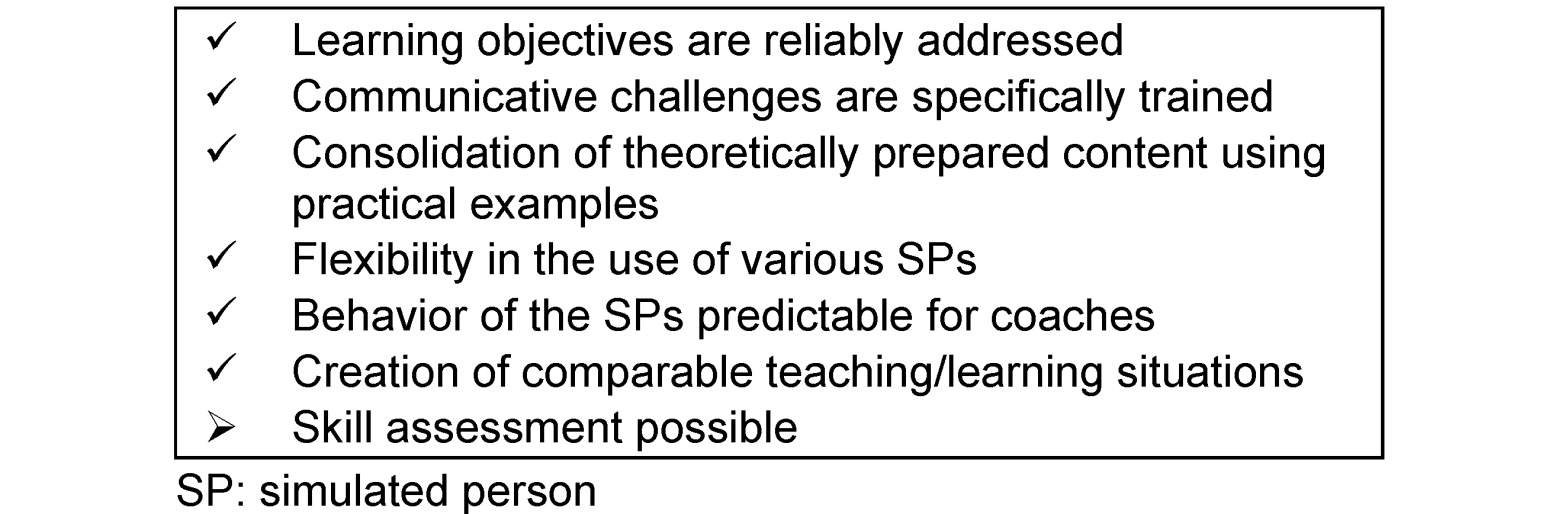
Benefits of standardised SP at a glance

**Table 3:**
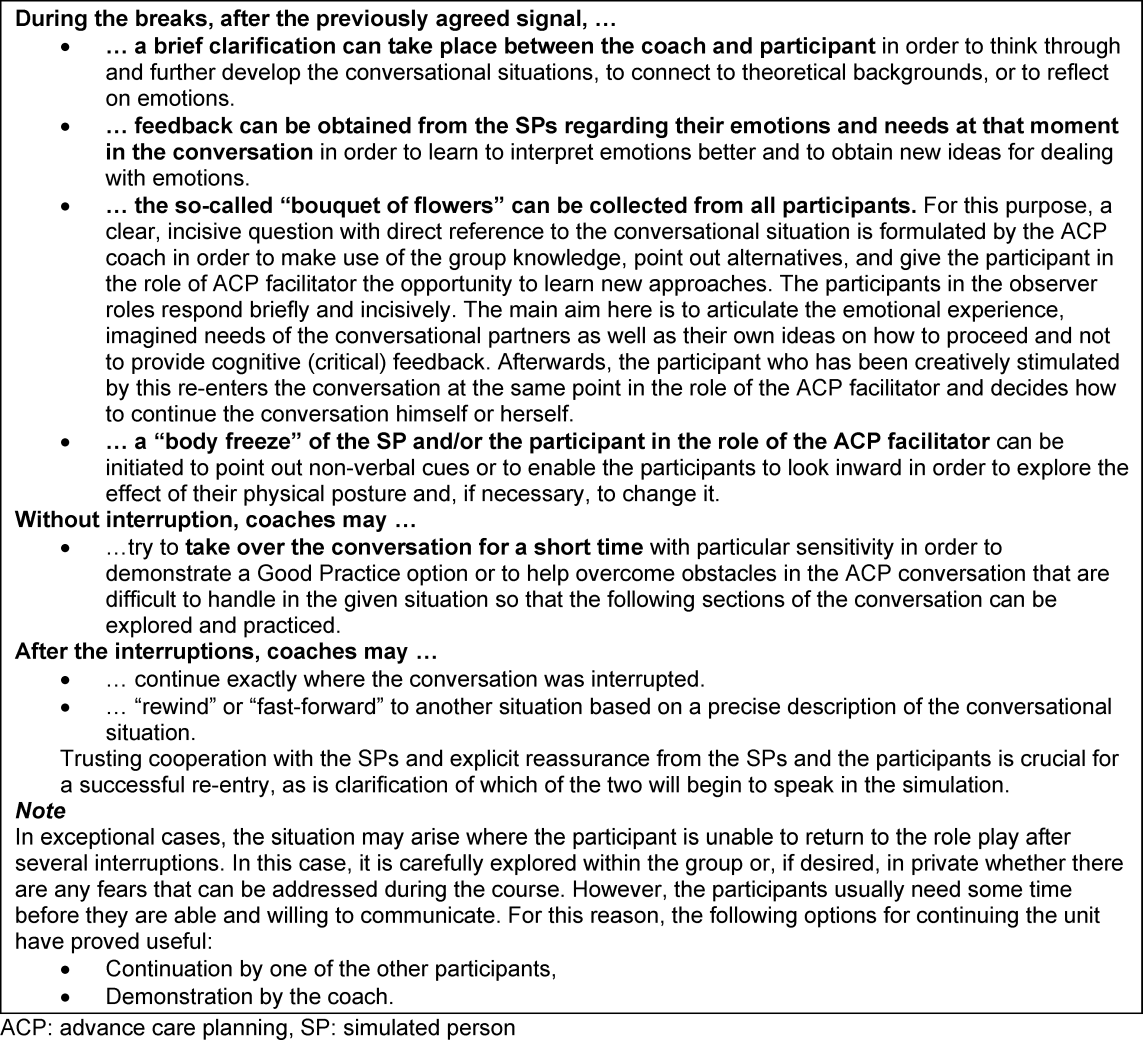
Techniques of microteaching
