# Supplementary material for: Training supported by simulated persons to promote the development of specific communication skills in advance care planning
Source: GMS J Med Educ. 2025 Feb 17;42(1):Doc11. doi: 10.3205/zma001735 (PMC12086250; doi:10.3205/zma001735)
Supplement: Course descriptions [file JME-42-11-s-001.pdf]

Attachment 1: Selected contents and formats of the ACP facilitator qualification

Methodology	before implementing SP	after implementing SP
Demo role-play	4	4
Participant role-play	11	11
SP-RP	0	24
Coaching regarding ACP-documentation after the course	20	7
Coaching regarding ACP-documentation between the 3 course modules	0	12
Supervision of conversations with real persons planning in advance	2	4

Demo: demonstration by ACP coach, SP: simulated person

Table S1A: Training opportunities as part of the ACP facilitator qualification before and after the implementation of SP-supported training in 2017 with details on the teaching lessons (45 minutes)

Attachment 1 to Götze K, Otten-Marré S, Loupatatzis B, in der Schmitten J. *Training supported by simulated persons to promote the development of specific communication skills in advance care planning.* GMS J Med Educ. 2025;42(1):Doc11. DOI: 10.3205/zma001735

Topic	Interactive Seminar	Demo role-play	Participants role-play	SP role-play
Introduction to medical indication and the patient's right to self-determination as the basis for the legitimation of medical treatment and further legal principles	2	0	0	0
Introduction to the concept of Advance Care Planning and the advance directive as an instrument of self-determination	2	0	0	0
The section of the ACP conversation on *attitudes towards living, dying and serious illness* and its documentation	2	2/3	2	5
The section of the ACP conversation on preferences in the event of *an acute health crisis with an uncertain prognosis* and its documentation	1	2/3	2	5
The section of the ACP conversation on preferences in the event of *incapacity to give consent of unclear duration (in hospital)* and its documentation	4	2/3	3	5
The section of the ACP conversation on preferences in the *event of permanent incapacity to consent* and its documentation	1 1/3	2/3	2	5
Tasks and selection of a legal proxy	2/3	0	0	0
The ACP facilitation with the proxy of a person unable to give consent and its documentation	1	1	2	4
Institutional und regional implementation of ACP	1	0	0	0
**SUM of lessons**	**15**	**4**	**11**	**24**

RP: role-play, P: participants of facilitator qualification, SP: simulated person. Note: The respective number of lessons may vary slightly between the individual course centers.

Table S1b: Selected contents and formats of the face-to-face part of the ACP facilitator qualification with specification on the amount of teaching lessons (45 minutes). The three modules of the qualification have a total of 72 teaching lesson. The content is deepened in further teaching lesson and in the coaching and supervision sessions (20 lessons).
