# Supplementary material for: Training supported by simulated persons to promote the development of specific communication skills in advance care planning
Source: GMS J Med Educ. 2025 Feb 17;42(1):Doc11. doi: 10.3205/zma001735 (PMC12086250; doi:10.3205/zma001735)
Supplement: Competencies of ACP facilitators according to structural, process and outcome criteria [file JME-42-11-s-002.pdf]

Attachment 2: Competencies of ACP facilitators according to structural, process and outcome criteria

Competencies of ACP facilitators according to structural, process and outcome criteria (slightly modified according to: Task Force "Advance Care Planning" of the German Society for Palliative Medicine (funded by the Federal Ministry of Health; 2017, unpublished).

1. Structural criteria:

- The ACP facilitators prepare the ACP conversations independently.
- The ACP facilitator meets the person planning in advance with an attitude of respect, empathy, openness and inquiring curiosity. The facilitators are guided by the desire and interest to support the person planning in advance in overcoming his/her misunderstandings and uncertainties, emotional barriers as well as barriers of unawareness and social desirability in order to identify their goals of care, determine their preferences and determinations.
- They use the tools of facilitation sensitively and responsibly and document the results of the conversation in a meaningful and explicit way.
- The ACP facilitators support the persons planning in advance and their loved ones and, if necessary, the institutions involved, by filing in the documents appropriately and making them available at any time, if required.

2. Process criteria:

- The ACP facilitators guide the course of the conversation in a trusting, sensitive and empathetic manner and facilitates the conversation in an open-ended manner, consistently focusing on and emphasising the attitudes, goals, wishes, needs and preferences identified during the course of the conversation.
- By using precise language that is easy to understand for the person planning in advance, the ACP facilitators ensure that the ACP relevant topics and clinical scenarios are precisely captured and made transparent for the person planning in advance. In this way, they ensure that the person making the advance care plan has an imagination of the situation that is as congruent as possible while contemplating on the situations which form - in technical terms - the basis of the written documentation of goals of care and treatment preferences.
- The ACP facilitators are sensitive to discrepant or ambivalent preferences; they identify and address inconsistencies and a lack of congruence in the content of individual statements during the course of the conversation. They open up a protected space of relationship in which the people planning in advance can reflect their goals of care and preferences and clarify as far as possible any remaining ambivalences in an exchange with the ACP facilitator and, if given, with participating confidants.
- The ACP facilitators substantiate the individual additions based on the needs and preferences of the person planning in advance in writing and incorporating them coherently into the advance directive.
- They formulate the results of the advance care planning discussion in an action-guiding manner and is appropriate for the recipient.

3. Outcome criteria:

- The ACP facilitators help the people planning in advance to gain clarity about their preferences, ambivalences and treatment goals in the various situations of future treatment for the event of decisional incapacity.
- The ACP facilitator document the statements, preferences and wishes substantiated in the course of the conversation in a manner that is clear, meaningful and action-guiding, even for persons not involved in the conversation.
- The attending physicians are integrated into the advance care planning process by the ACP facilitator, insofar as they allow this.
- The treatment goals and preferences of the person making the advance care plan are documented by the ACP facilitator in such a way that they form the basis for future updates of the preferences and, due to their informative value and validity, can be taken into account in the event of a crisis associated with decisional incapacity.
- The ACP facilitator help to ensure that the representatives know and appreciate the treatment goals and preferences of the person planning in advance and can represent them to treatment teams if necessary.
