# Supplementary material for: Training supported by simulated persons to promote the development of specific communication skills in advance care planning
Source: GMS J Med Educ. 2025 Feb 17;42(1):Doc11. doi: 10.3205/zma001735 (PMC12086250; doi:10.3205/zma001735)
Supplement: Competence and performance evaluation form according to the standards of ACP Germany [file JME-42-11-s-003.pdf]

Attachment 3: Competence and performance evaluation form according to the standards of ACP Germany

Competence and performance evaluation form according to the standards of ACP Germany

The following pages show the competence and performance assessment forms that have been developed to provide participants with structured summative feedback and to provide a sound answer to the question of whether the certification level (= passing level) has been achieved.

Bring the evaluation forms to the trainers' final conference!

ACP facilitation assessment | Module A / B (AD)

Fac.	Trainer:	Date	Place	Day: 1 2 3	Part./ SP / on Site	Role:

AD=advance directive; fac.= ACP facilitator; Part.=participant role-play; SP=simulated-people role-play; on site = on-site coaching; role=role of SP.

1	red	2	orange	3	yellow	4	Light green	5	green	na	Not applicable

Relevant emotions ("disturbances") GLOBAL:						
Relevant emotions recognized						
Relevant emotions named and endured						
Emotional "disturbance" cleared and conversation continued well						
General conversation techniques GLOBAL:						
Person-centeredness (according to C. Rogers): authenticity | empathy | caring						
Comprehensible information, appropriate differentiation						
Deepening through exploration (narrative stimuli)						
Red thread, priority for disturbances, re-focusing						
Open-endedness to results, minimization of suggestion / manipulation (attitude & technique)						
AD section attitudes towards living, dying and serious illness GLOBAL						
Start, expectations, focus, common platform.						
Elicit the person's individual attitudes						
How much does the person enjoy living, how strongly does the person wish to live on?						
What does the person think of dying? "Dying tonight?"						
May medical treatment help prolong life?						
Are there situations in which the person no longer ...?						
AD section FeNo GLOBAL						
Clarify situation/scenario, successful thematic focus.						
Clarify goal of care (AB vs. C) while respecting the individual's attitudes						
"Target options"/measures to be weighed and determined in accordance with the individual's attitudes						
A/B/C: favoured option vs. neighbouring option						
B: risks and burdens assessed acceptable						
B: medically accurate discussion of risks vs. chances						
Partial summary for this section including reference to documentation						
AD decisional incapacity of unclear duration GLOBAL						
Clarify situation/scenario, successful thematic focus.						
Clarify goal of care (AB vs. C) while respecting the individual's attitudes						
"Target options" / states to be weighed and determined in accordance to the individual's attitudes						
A/C: differentiation from B						
B: Will of the person planning in advance on occurrence of the undesirable situation						
B: accurate discussion of risks vs. opportunities.						
Partial summary for this section including reference to documentation						
AD permanent decisional incapacity GLOBAL						
Clarify situation/scenario, successful thematic focus.						
Clarify goal of care (AB vs. C) while respecting the individual's attitude						
"Target options" / states and measures to be weighed and determined in accordance to the individual's attitudes						
A/C differentiation from B (expand prior understanding of the person planning in advance, e.g., of the "faces of dementia")						
B: Criteria for changes in goal of care						
B: Further exclusion of specific medical measures						
Partial summary for this section including reference to documentation						
Feedback understood and accepted						
**Global score of this unit:** (no sum score)	1	2	3	4	5	X

Take Home Message: (use reverse side if necessary)

ACP facilitation assessment | Module C (AD-by-proxy)

Fac.	Trainer:	Date	Place	Day: 1 2 3	Part./ SP / on Site	Role:

AD=advance directive; fac.= ACP-facilitator; Part.=participant role-play; SP=simulated-people role-play; on site = on-site coaching; role=role of SP.

1	red	2	orange	3	yellow	4	Light green	5	green	na	Not applicable

	1	2	3	4	5	na
**Relevant emotions ("disturbances") GLOBAL:**						
Relevant emotions recognized						
Relevant emotions named and endured						
Emotional "disturbance" cleared and conversation continued well						
**General conversation techniques GLOBAL:**						
Person-centeredness (according to C. Rogers): authenticity | empathy | caring						
Comprehensible information, appropriate differentiation						
Deepening through exploration (narrative stimuli)						
Red thread, priority for disturbances, re-focusing						
Open-endedness to results, minimization of suggestion / manipulation (attitude & technique)						
AD-by-proxy section attitudes GLOBAL						
Start, expectations of proxy (p.), focus, common platform						
Creation of a sufficient information base						
Clarification of the proxy's task; separation of the represented person's will from the proxy's wishes						
Determining the individual (presumed) attitudes of the person represented (rp)						
How much does the person enjoy living, how strongly does the person wish to live on?						
What does the person think of dying? "Dying tonight?"						
May medical treatment help prolong life?						
Are there situations in which the person no longer ...?						
AD-by-proxy section FeNo GLOBAL						
Clarify situation/scenario, successful thematic focus.						
Clarify presumed goal of care of rP (AB vs. C) while respecting the rp's attitudes						
"Target options"/measures to be weighed and determined in accordance with the rp's attitudes						
A/B/C: favoured option vs. neighbouring option						
B: risks and burdens judged acceptable for rP						
B: medically accurate discussion of risks vs. opportunities						
Partial summary for this section including reference to documentation						
AD-by-proxy section Further orientation GLOBAL						
Clarify situation/scenario, successful thematic focus.						
Clarify presumed therapy goal of rp (AB vs. C) while respecting the rp's attitude and FeNo						
"Target options" / states and measures to be weighed and determined in accordance with the rp's attitudes						
A/C: differentiate from B (expand prior understanding, e.g. „Differing faces of dementia ")						
B: Criteria for changes in goals of care						
B: Determination regarding artificial nutrition						
Partial summary for this section including reference to documentation						
Feedback understood and accepted						

Global score of this unit: (no sum score)	1	2	3	4	5	X

Take Home Message:

From the very beginning of the facilitator qualification, there is a continuous accompanying assessment by the ACP coaches. The respective individual evaluation is not to be understood as a performance evaluation of the fully trained ACP facilitator in the sense of a final evaluation, but instead it helps by using a synopsis of all these snapshots to describe the potential and development of the participant during the course to achieve the ACP facilitator qualification in the end.

Scaling for the performance evaluation in the role plays / conversations (front)

Please use the following scoring key after each TN or SP role play:

**1**	Almost no essential tasks of the sub item (line) / subsection were solved satisfactorily; almost all aspects were missing and could not be implemented satisfactorily, if necessary, even after critical feedback.
**FB**	Could not understand or accept feedback.
**2**	Individual essential tasks of the sub item/subsection were satisfactorily accomplished; however, most essential aspects were missing and could not be satisfactorily accomplished, if necessary, even after critical feedback.
**FB**	Could understand and accept feedback with difficulty.
**3**	Some essential tasks of the sub item/subsection were satisfactorily completed; however, other essential aspects were missing and could not be satisfactorily implemented, if necessary, even after critical feedback.
**FB**	Was able to understand and accept feedback satisfactorily.
**4**	The essential tasks of the sub item/subsection were satisfactorily solved; critical feedback on missing aspects could be satisfactorily implemented, if necessary.
**FB**	Was able to understand and accept feedback well.
**5**	All essential tasks of the sub item / subsection were solved satisfactorily independently.
**FB**	Was able to understand and accept feedback excellently.
**Na**	This sub item / subsection was not practiced and therefore cannot be evaluated.

Explanation of selected subsections:

GLOBAL - Scores (first line of each section)	*no sum score of the subsection, different weighting of individual points possible.*
Getting started, expectations, focusing, common platform Common platform	*means that after getting started, the interlocutors talk about the same topic at eye level*
Clarify goal of care (AB vs. C) while respecting attitudes	*Goal of care to prolong life (possibly with limitations) vs. allow dying, while respecting the attitudes of the respective person*
Take Home Message	*Which feedback did you as a coach gave to the participant?*

Scaling for the assessment of suitability as a ACP facilitator (Coaches final conference)

For use in the ACP coaches' final conference at the end of each course module, taking into account the individual evaluation sheets from all training sessions as well as the global ACP coaches' impressions:

**1**	The trainee's suitability for the facilitator-role is not evident. Continuation is not recommended.<br>It is recommended to consider another role / task than the facilitation and not to continue the course qualification with the aim to work as a facilitator later on.
**2**	The trainee's suitability for the facilitator-role is unlikely. Continuation is not recommended.<br>It is recommended to consider a different role / task than the facilitator-role and not to continue the qualification with the aim to work as a facilitator later on.<br>Otherwise, extraordinary commitment and stamina as well as the ability to receive feedback, a considerably prolonged practice phase as well as increased coaching support through probably more than 6 individual coaching sessions in real conversations (or even a repetition of the entire workshop) appear to be preconditions for a successful completion of the qualification as facilitator.
**3**	The suitability of the trainee for the role of facilitator is in question. The continuation of the qualification can be supported (if desired by the trainee).<br>Intensive commitment and the ability to receive feedback as well as increased support of ACP coach through presumably up to 5 individual coaching sessions appear to be a precondition for successful completion.
**4**	The trainee's suitability for the role of facilitator is likely with continued commitment in the same manner. Continuation of the qualification is recommended.<br>The recommendations made in the feedback should be followed.
**5**	The trainee's suitability for the role of facilitator is given with continued commitment in the same manner. Continuation of the qualification is recommended.
