# Supplementary material for: Training supported by simulated persons to promote the development of specific communication skills in advance care planning
Source: GMS J Med Educ. 2025 Feb 17;42(1):Doc11. doi: 10.3205/zma001735 (PMC12086250; doi:10.3205/zma001735)
Supplement: Description of the challenges of the simulated persons (SP) roles and most relevant training objectives for the ACP facilitator qualification [file JME-42-11-s-004.pdf]

Attachment 4: Description of the challenges of the simulated persons (SP) roles and most relevant training objectives for the ACP facilitator qualification

SP role	Age (years)	AD/AD by proxy	Social situation	Medical situation
Mrs/ Mr G.	72	AD	Self-employed photographer, widow(er), 1 sister, living alone in her/his own flat	No previous illnesses
Mrs/ Mr. K.	86	AD	Retired (formerly self-employed), widowed, 3 children, 4 grandchildren, lives in a retirement home	Pneumonia with ventilation 2 yrs ago, consecutive long stay on intensive care unit and subsequent rehabilitation, osteoarthritis of both hips with walking disability.
Mrs/ Mr. K	82	AD	Pensioner (formerly Kindergarten teacher/tool mechanic), 2 children, 3 grandchildren, lives in a retirement home	Mild dementia, high-blood pressure, not insulin dependent diabetes mellitus type 2
Mrs/ Mr. K	76	AD-by proxy	Pensioner (formerly construction worker/meat salespersons), 1 son, 2 grandchildren, divorced, in new partnership, living alone. Conversation relates to ACP for ex-husband/wife for whom Mrs/Mr. K is the surrogate for: 81yrs, living in nursing home	Multiple severe strokes, since 10 yrs no longer capable of decision-making, high need of care.
Mrs R.	78	AD-by proxy	Retired (formerly worked in a workshop for the disabled), single, no children, parents deceased, no siblings	Congenital moderate cognitive impairment
Mrs/ Mr S	66	AD	Retired (formerly manager), married, 2 children, 3 grandchildren; Lives in her/his own house	History of stroke without neurological symptoms
Mrs/ Mr S	73	AD	Retired (former office administrator), divorced, 2 children, 5 grandchildren; living alone	Mild heart failure, advanced non-Hodgkin's lymphoma
Mrs/ Mr S	87	AD-by proxy	Retired (formerly private secretary in the family business), married, 1 son, 3 grandchildren, lives in nursing home<br>Conversation relates to ACP for spouse: 84 yrs. (former manager of the family business)	Dementia for 7 yrs, meanwhile severe
Mrs/ Mr S	60	AD-by proxy	Early retired teacher, surrogate of Mrs. R. Conversation relates to Mrs. R.	See role of Mrs. R.
Mrs/ Mr W	82	AD	Pensioner (formerly administrative employee), 1 daughter, 2 grandchildren, new to the retirement home	History of a fall with hip fracture caused by frailty, no life-limiting illness

Note: The roles are constantly evolving, and both the presentation and challenges/training objectives may change. *Caption:* ACP: advance care planning, AD: advance directive, AD-by proxy: advance directive by proxy, [19], yrs: years.

Table S2A: Key data of the roles for the simulated persons

Roles	Challenges	Training objectives: The GB …
Role 1	Good QoL regardless of advanced cognitive impairment, with a desire to continue living in the current state for a long time.<br>Can no longer articulate preferences regarding possible limits of future life-sustaining treatments due to dementia. The memory of previous vehement statements made by the person who is unable to give consent with regard to medical treatment makes it difficult for the surrogate to reconstruct the presumed will taking into account the current circumstances and statements.	... keeps the perspective of the person being represented in focus without ignoring the surrogate with his/her own emotions and challenges.<br>... recognises, addresses and supports the surrogate in recognising misunderstandings, emotions and possible own wishes, and putting them aside with the effect that ACP in accordance with the (presumed) wishes of the represented person becomes possible.
Role 2	Enjoys living, but not at any price. Witnessed courses of illnesses of good friends lead to the wish for radical limitations of future medical treatments. However, these preferences are complicated by misunderstandings.	… recognises the person's misunderstandings (which is frequently encountered in planning for the scenario "acute emergencies with incapacity to consent") and supports the person in clarifying these misunderstandings in order to be able to make a valid determination.<br>…anticipates the person's limited understanding of the multifaceted condition of "permanent loss of decision-making capacity", recognises the associated prejudices and dissolves them cautiously and gradually so that the person can eventually arrive at an informed decision.
Role 3	The person who is not able to give consent has a protective maternal/paternal relationship with the surrogate for that has grown over decades. Caring for him or her has become an important part of his or her life.<br>The close relationship makes it difficult to realise that the person represented is becoming more fragile and withdrawing.<br>In addition, personal experiences cloud the view of future medical treatment in the event of the represented person becoming seriously ill.	... cautiously but consistently introduces the surrogate to considerations and insights that he/she is reluctant to reflect on, even though they are essential for advance care planning by proxies.<br>... reflects whether ACP is currently possible in view of the given resistance on the side of the surrogate (but when would it be possible?) and respects the limits indicated while at the same time helping to appreciate the perspective of the person being represented.<br>... endeavours to enable the surrogate through careful guidance and suggestions to gain a realistic picture of the presumed preferences of the person being represented.<br>... encourages the surrogate to put aside his/her own agenda, and thus to engage in ACP in the presumed interests of the represented person.<br>... helps to reflect on medical treatment options with regard to the personal circumstances and needs, in order to be able to plan in advance on an individual basis.
Role 4	A timid person, seeking to fulfil social expectations in contact with family and healthcare providers.<br>Timidness and attention to social expectations of others set aside, the person prefers a palliative goal of care regardless of the current high QoL and of an acceptable prognosis in case of a life-threatening event.	…enables the development of a trust-based relationship.<br>…displays a genuine, strong interest in the person and a non-judgmental openness for his/her individual attitudes and treatment preferences, so that the person can overcome his/her obstacles and reservations and communicate authentically.
Role 5	Reluctant to communicate because intimate experiences are touched. Strong desire to live (combined with a fear of dying) in the face of a life-limiting disease. The desire to live originates from a newly gained QoL, therefore willing to take high risks and burdens in order to safe this life that has become precious.<br>Smouldering fears make it difficult to take a realistic view on the ACP scenarios and a valid ACP process.	…supports the person in becoming aware of, reflecting on and channelling his/her strong emotions regarding the wish to live and the fear of dying in order be able to open up for the ACP process.<br>…leads through the conversation with openness and sensitivity, so that the person can deal with ACP questions he/she considers relevant<br>…addresses the low prognosis considering the severe underlying disease of the person .

Roles	Challenges	Training objectives: The GB …
Role 6	Little is known about the care preferences of the person who is now incapable of giving consent. The surrogate is characterised by uncertainty, anxiety and pronounced social desirability, but at the same time he/she is the only person who knew the incapacitated person before his/her cognitive impairment.<br>According to the assessment of the nursing staff, the represented person's QoL is poor, even perceived as suffering/agony, yet life-sustaining treatment courses were repeatedly taken in health crises.	…understands and respects the possibilities, challenges and limitations of the surrogate decision maker.<br>…enables and encourages the primarily insecure surrogate to reflect on the ACP issues without exerting pressure or ignoring ambivalences.<br>…identifies and weighs the existing evidence of the presumed preferences of the incapable resident, involving the surrogate in the critical judgment as to whether this evidence is sufficiently reliable for a determination in the sense of advance care planning.
Role 7	Closed, arrogant, disappointed and angry, answers in monosyllables, sometimes in generalisations, likes to take the lead in the conversation. The background is a serious disappointment experienced in the recent past. Depending on the course of the conversation, escalates or attempts to delegate the determination for care preferences to the facilitator with resignation.	... recognises countertransference phenomena and uses them to understand the person planning in advance and the resulting conduction of the conversation.<br>... reacts empathetically to anger and aggression and enables the person to express his/her central emotions and thus to gain distance from them in order to be able to do ACP in a valid way.
Role 8	A woman with limited vitality, incapable of giving consent due to congenital moderately severe cognitive impairment. She has withdrawn in recent years as a result of various experiences and increasing fragility. Defines herself by her tasks in the community, but has withdrawn over the last few years. Great trust in the surrogate.	... recognises the possibilities, challenges and limits of assisted self-determination.<br>... seeks personal contact with the incapable person and makes an attempt to reach an assisted decision before continuing the ACP conversation with the surrogate.
Role 9	Intensive desire to live and good QoL regardless of moderate dementia restrictions. Has a lot of trust in life and the people around her including strangers.<br>Cognitive impairments are a challenge when dealing with complex issues.<br>Long stretches of speech or complicated terms on the part of the ACP facilitator risk losing the person.<br>Strong fears regarding a hypothetical scenario in the event of serious illness.	... understands the possibilities, challenges and limits of assisted self-determination.<br>... can express complex issues of advance care planning in simple language.<br>... overcomes own barriers of anxiety in order to sensitively and openly address the person with mild or moderate dementia with regard to probable future cognitive deterioration.<br>... compensates for unexpected strong emotions of the person with dementia, which may limit the ability to plan in advance.
Role 10	Cheerful, active person who accompanied his/her spouse when he/she died a few years ago. In this context, certain conceptions have become solidified that make it difficult to plan in advance consistently.	... elicits diverse experiences of the person making an advance care plan, structures them and organises them in the context of advance care planning.<br>... recognises and addresses inconsistent attitudes and preferences and enables the person planning in advance (if possible and desired) to gain greater clarity and develop consistent preferences for ACP.

ACP: advance care planning, QoL: quality of life

Table S2b: Challenges and training objectives of the simulated persons roles

Only the most relevant challenges and associated exemplary training objectives are described in the table. The training objectives mentioned in the table relate to the specific skills needed in the respective roles, not the basic competencies (ACP-specific knowledge, attitude, structured facilitation of the four sections of the ACP process) which are practiced during all role-plays. Some roles are designed to be extra simple so that they should not challenge the participants beyond the basic competences.
Note: The roles are constantly evolving, and both the presentation and challenges/training objectives may change. The order of the roles is arbitrarily reversed to table 2a so that potential course participants may not easily assign them.
Section 1: Attitudes towards life, dying and serious illness (AD and AD-by proxy), *Section 2:* ACP for an acute emergency (AD and AD-by proxy), *Section 3a:* ACP for the event of an in hospital treatment for the situation of decisional incapacity of unclear duration (AD only), *Section 3b*: ACP for the event of a chronic deterioration of the represented person’s condition (AD-by proxy only), *Section 4:* ACP for the event of permanent decisional incapacity (AD only)

Attachment 4 to Götze K, Otten-Marré S, Loupatatzis B, in der Schmitten J. *Training supported by simulated persons to promote the development of specific communication skills in advance care planning.* GMS J Med Educ. 2025;42(1):Doc11. DOI: 10.3205/zma001735
